# Formulations, Processing, and Application of Poly(butylene adipate-co-terephthalate)/Thermoplastic Starch Blends: A Review

**DOI:** 10.3390/polym17111457

**Published:** 2025-05-23

**Authors:** Aline N. Küster, Cidalia Paula, Juliana Azevedo, Arménio C. Serra, Jorge F. J. Coelho

**Affiliations:** 1 Department of Chemical Engineering, University of Coimbra, CEMMPRE, ARISE, Rua Sílvio Lima, Pólo II, 3030-790 Coimbra, Portugal; alinekuster@gmail.com (A.N.K.); aserra@eq.uc.pt (A.C.S.); 2 Componit, Estrada Real 3, 2070-621 Vila Chã de Ourique, Portugal; 3 Compo-star, Estrada Nacional N.º 3, Km 16, Casais da Lagoa, Aveiras de Baixo, 2050-038 Lisboa, Portugal; juliana.azevedo@compostar.eu; 4 IPN, Instituto Pedro Nunes, Associação para a Inovação e Desenvolvimento em Ciência e Tecnologia, Rua Pedro Nunes, 3030-199 Coimbra, Portugal

**Keywords:** PBAT, TPS, starch, bioplastics, packaging

## Abstract

The concern for the environment and sustainability has intensified the search for alternative materials to replace non-degradable plastics. Poly(butylene adipate-co-terephthalate) (PBAT) is a bioplastic that has been extensively studied due to its excellent mechanical properties, which are similar to those of low-density poly(ethylene) (LDPE). However, the high cost of this polymer still hinders its wider application. Among the different approaches that have been studied, blending PBAT with thermoplastic starch (TPS) could be an interesting solution to reduce the cost of the material and increase the degradability of the blends. This review covers most of the work reported in recent years on PBAT/TPS blends, including the effects of starch plasticizers, starch modifications, processing methods, use of chain extenders, various compatibilizers, and additives used for different applications.

## 1. Introduction

Due to their versatility, low cost, light weight, and other properties, the consumption of plastics is increasing every year [1,2]. However, concerns over high CO_2_ emissions and the lack of proper end-of-life management system have led to the search for alternative bio-based and/or biodegradable materials, known as bioplastics [2,3]. Bioplastics encompass a range of materials with different properties and applications, and today there are already alternative bioplastics on the market for almost every conventional plastic [2]. Global bioplastics production capacity is expected to triple between 2021 and 2026 [4].

PBAT is a fully biodegradable and flexible copolyester derived from fossil resources [5,6,7]. Its mechanical properties are comparable to those of LDPE, one of the most commonly used plastics [5,6,8]. In addition, it has suitable barrier properties for food packaging and is approved for food contact applications [6,7]. However, its high production costs compared to the traditional fossil-based polymers and limited thermomechanical properties restrict its use in a wide range of applications [5,7,9]. Blending PBAT with plasticized starch can be an effective alternative to reduce the cost of PBAT-based materials for a wider range of applications [7,10].

Starch is a carbohydrate derived from renewable sources, making it an extremely cost-effective polymer [7]. It is composed of amylose, a linear amorphous polymer, and amylopectin, a highly branched crystalline polymer. When starch is used in a composite material, it is in a granular state due to the hydrogen bonds between amylose and amylopectin, which leads to insufficient dispersion within the matrix and affects the properties of the final product [11]. In addition, the melting temperature range of starch overlaps with the onset of its degradation [12,13,14]. Therefore, a common approach to improve the interaction between the thermoplastic matrix and the starch is therefore to plasticize the starch under heating with a suitable content of water and plasticizers to convert it into TPS [6,11]. The addition of plasticizers leads to a gelatinization of the starch, which consists in a disruption of its crystalline structure. The content and combination of additives can be varied to optimize the processing properties of TPS [13]. In order to increase the interfacial adhesion and TPS content in PBAT/TPS blends while maintaining the balance between stiffness, strength, and elongation at break, a compatibilizer should be added to the mixture [7,15]. Various compatibilizers have been reported in the literature, such as citric acid, soybean oil, maleic anhydride, nanocellulose, and tartaric acid [6,7,16,17]. Generally, a reactive extrusion is used for the compatibilization strategy, which allows functional groups to be grafted into the polymer chains quickly and continuously [17].

In recent years, blends of PBAT and TPS have been extensively researched in the literature. Numerous studies have investigated various processing techniques, compatibilizers, and additives to optimize the properties of these blends, primarily for the development of biodegradable films, especially for food packaging. Despite this focus, PBAT/TPS blends hold significant potential for replacing conventional plastics in a wider range of applications. Given the growing body of research and the diversity of formulations and strategies used, a comprehensive and structured overview is timely and valuable. This paper aims to summarize the key findings on PBAT/TPS blends and provide an overview of PBAT and starch/TPS properties, common processing methods, starch modifications, the role of chain extenders, various compatibilizers, ternary systems, additives for active packaging, and new applications of these materials.

## 2. PBAT

**Figure 1:**
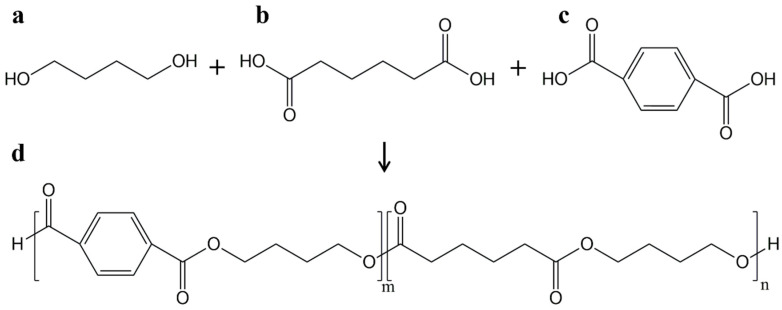
PBAT synthesis. (**a**) 1,4-butanediol; (**b**) adipic acid; (**c**) terephthalic acid; (**d**) PBAT.

PBAT is an aliphatic–aromatic copolyester based on raw fossil materials [2,5]. It can be produced by polycondensation of 1,4-butanediol with adipic and terephthalic acid (Figure 1) [2,18]. Zinc acetate and rare earth compounds can be used as catalysts. The polyesterification requires a long time (~19 h), a high vacuum, and temperatures usually above 190 °C. These conditions are necessary to favor condensation reactions and to remove the products [2,19].

PBAT is a semi-crystalline thermoplastic polyester that is flexible and completely biodegradable. Its mechanical properties are comparable to those of LDPE [2,7]. It is also approved for food contact applications and has interesting barrier properties for food packaging compared to LDPE, such as low oxygen permeability and high water vapor permeability [5,7]. However, the high production costs, which are about three times higher than those of LDPE, and the low thermomechanical properties limit its use in a wider range of applications [2,5].

The mechanical properties of PBAT depend on the terephthalate content and the molecular weight [2]. For example, the maximum terephthalic acid content that improves the mechanical strength while maintaining the biodegradability of the material is about 40 wt% [18]. Since the pressure and temperature of the reactor can shift the reaction towards the products and influence the PBAT molecular weight, the mechanical properties of the final polymer can be tailored by adjusting the process variables [2].

Another method to improve the final mechanical properties of PBAT is the use of chain extenders in the high-temperature molding and processing stages. However, this approach can lead to crosslinked polymers with undesirable properties and limited recyclability. For example, the leading developer and manufacturer of PBAT, BASF, uses glycerol as a branching agent in the production of ECOFLEX^®^ PBAT [20].

The addition of fillers is also useful to improve the final performance of the polymer and reduce material costs while maintaining biodegradability. There are three main methods for producing this type of composite: in situ polymerization, melt blending, and solvent casting. The most common methods for producing these PBAT-based composite materials are melt blending, extrusion, or injection molding. A high shear process is needed to promote the dispersion and distribution of the reinforcing materials and to enable large scale production. However, due to the high viscosity and non-polar nature of PBAT, the dispersion of hydrophilic fillers can have a negative effect and reduce the mechanical properties of the final material. Some solutions to this problem have been described in the literature, as the chemical modification of fillers to reduce their hydrophilicity [2].

The literature reports the use of various fillers to produce PBAT-based composites, such as cellulose nanocrystals, montmorillonites, and natural fibers. Currently, PBAT-based composites are used for various purposes, such as food packaging, biomedical, and environmental applications [2].

## 3. Starch

Starch is a polymeric carbohydrate derived from renewable sources, making it an extremely cost-effective polymer [5]. The size, morphology, surface properties, internal structure, and other physicochemical parameters of starch vary from source to source, and there are many producing plants that are not yet well characterized [21]. The most commonly used starch sources are maize (almost 80%), cassava, wheat, and potatoes [22].

**Figure 2:**
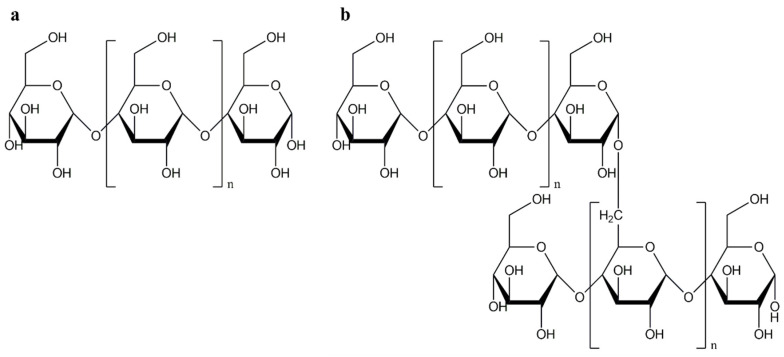
Molecular structures of (**a**) amylose and (**b**) amylopectin.

It consists of amylose and amylopectin, a linear amorphous and a highly branched crystalline polymer, respectively (Figure 2). The proportions of amylose and amylopectin are usually 20–25% and 75–80%, respectively, depending on the source [23]. There are some varieties of rice, maize, and barley that reach an amylopectin content of 100% and are described as waxy [24]. In addition to amylose and amylopectin, starch also contains other minor components such as lipids, proteins, and minerals [22].

Since starch can have different amylose contents, there are significant differences in the mobility of the polymer chains, resulting in different mechanical and thermal properties. It has been reported that starch with high amylose content forms films that are more transparent, colorless, and have low oxygen permeability [25]. Zhang et al. prepared PBAT/modified starch films using four starches with different amylose contents. They showed that with a higher amylose content, the crystallinity of the films increased, which is due to the interaction of the amylose helices forming semi-crystalline units [25].

Some of starch properties, such as hydrophilicity and poor mechanical properties, hinder the direct use of starch [26]. In addition, native starch is in the form of granules due to the hydrogen bonds between amylose and amylopectin, and this granular state leads to insufficient dispersion in the matrix, which affects the properties of the final product [14]. In addition, the melting range of starch (220–240 °C) overlaps with the onset of its decomposition (~220 °C) [12].

A widely used approach to overcome these disadvantages is to plasticize starch with an appropriate amount of plasticizer under heat and shear to convert it into thermoplastic starch [14]. The plasticizer cleaves the hydrogen bonds between the starch molecules and partially depolymerizes the starch polymer, resulting in melting and glass transition temperatures below the decomposition temperature [18]. The processing properties of TPS can be adjusted by varying the content and combination of additives [27].

Due to its high sensitivity to moisture, high viscosity and high tendency to biodegrade, the mechanical properties, processability and shelf life of TPS can be impaired, which limits its application possibilities [5]. Thus, TPS is usually blended with another polymer, which should ideally be hydrophobic, to prevent moisture uptake and to enhance the mechanical properties [26].

## 4. PBAT/TPS Blends

**Figure 3:**
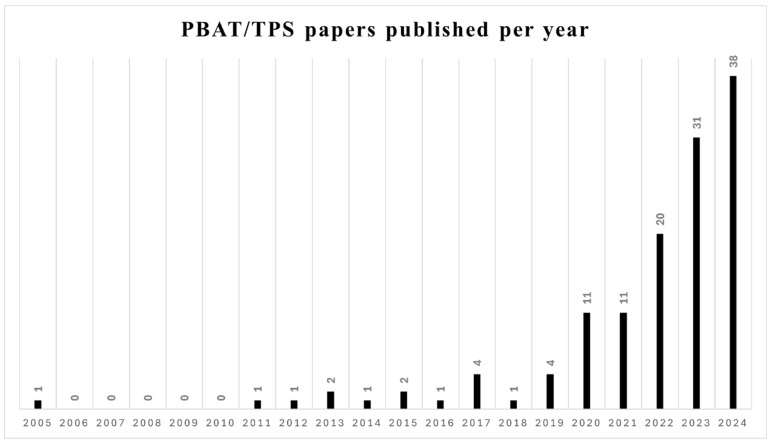
Number of papers on PBAT/TPS published each year (data from PubChem 2024 include papers published until November 2024).

Since 2020, the number of studies on PBAT/TPS blends has increased significantly (Figure 3). Researchers are particularly interested in these polymers as their blending results in a material that has similar properties to commercial plastics but has the advantage of being fully biodegradable. Most studies have focused on compatibilizers to increase the interfacial adhesion between PBAT and TPS and consequently improve their mechanical, thermal, and permeability properties. In this section, the processing parameters, PBAT and TPS content in the blend, starch modifications, compatibilizers and additives, ternary blends, active packaging, and other applications of the blends are discussed.

### 4.1. Processing Conditions

One problem with many biodegradable materials is that they exhibit relative thermal instability during processing, for example in comparison to polyolefins [7]. Therefore, it is important to investigate different processing parameters, such as the temperature profile and screw speed, and to select a range of parameters that lead to materials with optimal properties.

Lekube et al. investigated the effects of the processing conditions of the compounding and blown film extrusion processes on the properties of TPS/PBAT materials. In the compounding process, the temperature, screw speed, and throughput were varied, while in blown film extrusion, only the temperature and screw speed were changed [3]. During compounding, low specific throughputs led to smaller starch particle sizes and high values for elongation at break and tensile strength of the films, which can be explained by the stronger shear forces during dispersive mixing. The tear resistance and elastic modulus of the films showed optimum values at medium specific throughputs. The visual appearance of the films showed a degradation of the material at low specific throughput values. Lower temperatures lead to better tensile properties and more transparent (less degraded) films [3].

Variations in temperature and screw speed during blown film extrusion did not lead to significant changes in the mechanical properties of the films. However, the visual appearance of the blends processed at lower screw speeds and higher temperatures resulted in less coloration of the films. Thus, although no significant changes in material properties were observed in blown film extrusion by varying the processing conditions, the results in compounding show that changes in the processing parameters during this phase can strongly influence the material properties [3].

Biodegradable materials such as PBAT and TPS are likely to degrade under a strong shear flow field, resulting in poorer mechanical behavior. It has been reported that the use of an extensional flow field can promote more effective dispersion and breakage of the dispersed phase, resulting in better compatibilization. In addition, biodegradable materials are less susceptible to degradation under an extensional flow [28]. Xu et al. successfully produced PBAT/TPS blends using an eccentric rotor extruder dominated by an extensional flow field. The periodic compression and release of the volume between the stator and the eccentric rotor which make up the extruder creates the extensional flow field [28].

In addition to the processing equipment and parameters, there are also different methods for processing the blends. For example, Brandelero et al. investigated the effects of the production method on the properties of PBAT/TPS films. More specifically, they analyzed the difference between the extrusion of (previously prepared) TPS pellets together with PBAT pellets (method M1) and the joint extrusion of pelletized starch, plasticizer, and PBAT pellets (method M2). It was found that in method M2, the films with higher starch content (>50 wt%) had similar properties to the films with lower starch content. The authors explained that this is probably due to the larger contact area of granular starch compared to TPS pellets, resulting in a better interaction between the two polymers [29].

On the other hand, Bai et al. investigated the difference between a one-step and a two-step extrusion method (corresponding to methods M1 and M2 from the previous paragraph, respectively) in the preparation of PBAT/TPS blends with a reactive epoxy compatibilizer. Using the two-step extrusion method, the tensile strength, elongation at break, and impact strength values of the samples were ~47%, ~4%, and ~13% higher, respectively, compared to the samples produced using the one-step extrusion method. These improvements can be attributed to a morphological transition from a droplet-in-matrix structure to a more favorable bicontinuous phase-separated structure [30].

### 4.2. PBAT and Starch Content

Another way of influencing the material properties without the addition of compatibilizers or additives is to vary the PBAT and starch content in the mixture. The phase morphology of immiscible polymer blends, which is influenced by the blend content, has a significant impact on the mechanical properties of the material. Normally, the main component appears as a continuous matrix and the minor component is dispersed as spherical or fibrous structures in the matrix. The mechanical properties of this type of mixture depend on the continuous matrix and the morphology of the dispersed phase [31]. Moreover, starch not only degrades more than PBAT during processing, but also increases the degradation of PBAT [7]. Therefore, it is crucial to adjust the optimal ratio depending on the intended application.

Zhai et al. reported the effects of starch/PBAT weight ratio in nanocomposite films prepared using a one-step method in which starch, plasticizer, PBAT, and other additives were mixed simultaneously, without prior TPS formation. In films with 100 wt% starch, strong hydrogen bonds between the chains of starch molecules resulted in the difficulty of movement of these chains. By increasing the PBAT content, the strong hydrogen bonds between the starch molecule chains were destroyed by the separating effect of the PBAT molecules, which facilitates the movement of the molecular chains. The addition of PBAT therefore reduces the stiffness of the films. With the same PBAT and starch content, the structure of the surface improved to a smooth, dense, and uniform appearance without cracks, wrinkles, or irregularities. This coarser and ductile surface is indicative of improved interfacial adhesion between starch and PBAT. The addition of PBAT significantly improved the tensile strength and elongation at break of the films, especially above 30 wt% [32].

### 4.3. Starch Modifications

For many industrial applications, native starches do not have optimal physico-chemical properties; they are insoluble in water, largely inert, and highly resistant to enzymatic hydrolysis. Therefore, native starches are often modified to achieve better physical and functional properties [21]. There are various methods for modifying starch, including physical, chemical, and biological methods, or a combination of these methods. Chemical modifications include the introduction of functional groups by esterification, etherification, oxidation, or acid hydrolysis of starch [24]. Biological modifications involve the use of enzymes to hydrolyze starch, resulting in partially hydrolyzed or solubilized products. Physical modifications can be carried out by irradiation, ultrasound, extrusion, plasma, high pressure, electric fields, annealing, dry heating, and heat–moisture treatment [24].

Although physical methods have clear advantages, chemical modifications are preferred. These modifications aim to produce more hydrophobic products, and the most obvious point of chemical modification is the hydroxyl groups of the starch chains at positions C-2, C-3, and C-4 [26,33]. For example, starch acetylation is useful for extruded starch-based foams. However, this process leads to poor mechanical performance and high product costs [18]. Another starch modification is the phosphorylation of the hydroxyl groups in the anhydroglucose units, which leads to crosslinking, and thus to a strengthening of the hydrogen bonds between the starch molecules. This modification leads to a higher resistance to temperature, low pH value, and high shear forces [34].

**Figure 4:**
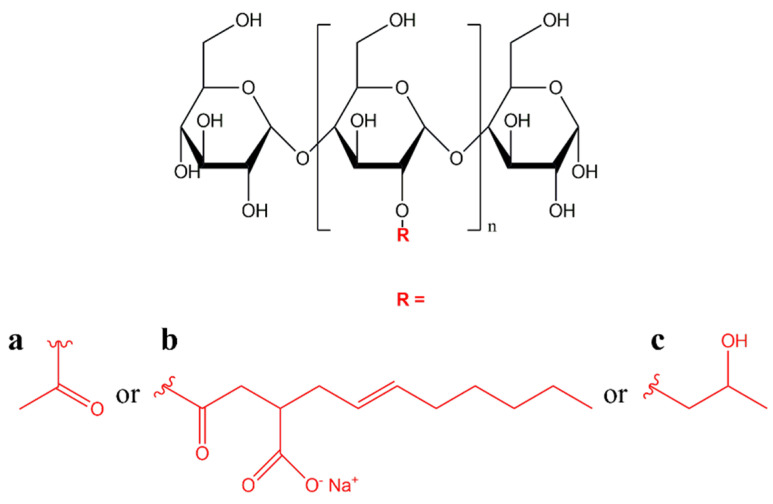
Molecular structures of modified starches. When R = (**a**), the result is acetylated starch; when R = (**b**), the result is octenyl-succinated starch; and when R = (**c**), the result is hydroxypropylated starch.

Wongphan et al. investigated the effects of using native or modified starch (acetylated, octenyl-succinated, or hydroxypropylated—Figure 4) in PBAT/TPS packaging materials. When octenyl-succinated starch was used, compatibility with PBAT was improved compared to the other starch types tested, which can be attributed to the increased hydrophobicity of the octenyl-succinate groups; however, the barrier and mechanical properties of the films were compromised. In contrast, native and hydroxypropylated starches were more hydrophilic, resulting in dispersed phases; and when the content of these starches increased, fiber-like TPS networks were formed, which became entangled in the PBAT [35].

Wadaugsorn et al. investigated the effects of the degree of substitution of the hydroxypropyl groups in blends of PBAT with TPS (from hydroxypropyl cassava starch) for the production of bioplastic packaging. They reported that increasing the degree of substitution of hydroxypropyl groups in TPS effectively improved the clarity of the films. The miscibility between PBAT and TPS was also improved by higher degrees of substitution, resulting in a co-continuous structure that gave more flexible films. In addition, a higher TPS content with a high degree of substitution improved the mechanical strength of the films [36].

### 4.4. Starch Plasticizers

Some of the plasticizers used in the production of TPS are glycerol, fatty acids, vegetable oils, triacetin, polyadipates, citrates, polysuccinates, polyethylene oxide, urea, formamide, and acetamide. Among them, glycerol is the most commonly used plasticizer due to its three hydroxyl groups that form strong bonds with starch [18,37]. However, after prolonged storage, starch plasticized with glycerol tends to recrystallize, making the material brittle. To prevent the retrogradation of the starch, plasticizers with amide groups, such as urea, enable stronger hydrogen bonds with starch than with polyols. The problem with these plasticizers with amide groups is their toxicity, which limits their use [37].

Ivanič et al. compared the use of urea and glycerol as starch plasticizers in PBAT/TPS blends. It was found that the blends plasticized with urea had a smoother surface, resulting in higher tensile strength and elastic modulus values, especially when the TPS content was 50 wt%. This is due to the fact that the hydrogen bonds formed between the amide groups of urea and the hydroxyl groups of starch are much stronger than those formed between glycerol and starch [38].

He et al. investigated the effects of using glycerol/citric acid/stearic acid or acetyltributylcitrate (ATBC) as starch plasticizers in PBAT/TPS blends. The films produced with 30 wt% glycerol as plasticizer showed improved compatibility between the polymers, resulting in better mechanical properties. The mechanical properties of these films were also better than those of the other samples after 48 months, which can be explained by the migration of glycerol from the TPS phase into the PBAT phase. On the other hand, the films with ATBC as plasticizer absorbed less water than the films with glycerol, which is probably due to the hydroxyl groups of glycerol compared to the ester bonds present in ATBC [12].

Due to plasticizer migration during the lifetime plasticized starch, it is important to choose a suitable plasticizer that provides improved plasticity and weight bearing capacity without migrating during storage. A deep eutectic solvent (DES) consists of a hydrogen bond donor (HBD) and a hydrogen bond acceptor (HBA), that are liquid at temperatures below their respective freezing points when combined. This solvent can be compatible with both hydrophilic and hydrophobic polymers. DES are therefore suitable plasticizers for starch in PBAT/starch blends [39].

Meng et al. prepared PBAT/starch films using a DES consisting of choline chloride as HBD and glycerol as HBA. DES was used to improve the degradation rate and mechanical properties of the PBAT/starch film. The increased hydrophilicity of starch and DES favored the proliferation of microorganisms, resulting in faster degradation of PBAT. The faster degradation is also due to the release of acid during DES degradation, which also breeds microorganisms. Compared to the films containing only PBAT and starch, the addition of 10 wt% DES resulted in an increase in tensile strength by ~8% and elongation at break by ~167%, as well as increased transparency of the films. These results can be explained by improved interfacial interactions between PBAT and starch [39].

### 4.5. Chain Extenders

Chain extenders have been widely used to obtain polymers with higher average molecular weights and to some extent to act as a blending compatibilizer [40]. However, this approach can also lead to crosslinked polymers with unpredictable properties and limited recyclability [20].

**Figure 5:**
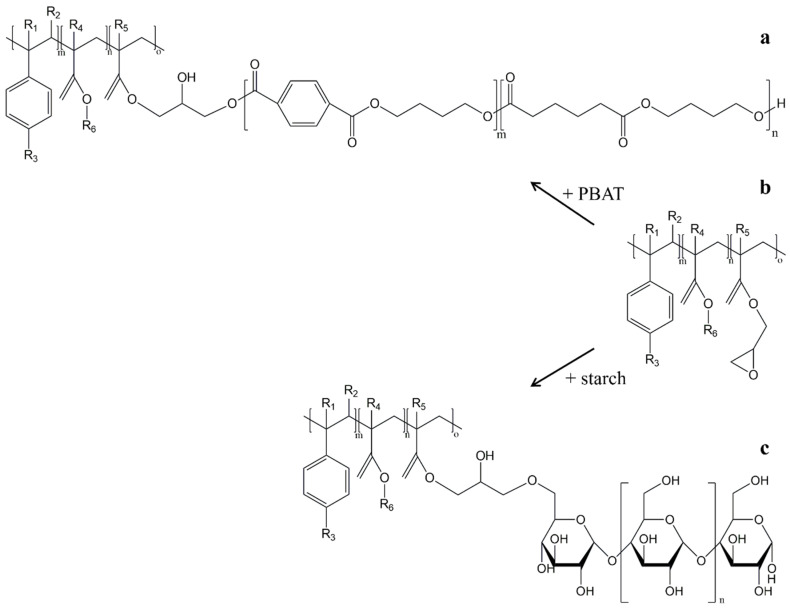
Molecular structure of commercial chain extender Joncryl-ADR-4368 (**b**) and possible reaction with PBAT (**a**) and starch (**c**). Adapted from [42].

One of the most commonly used chain extenders is Joncryl-ADR-4368, a commercially available polymer used to improve the properties of biodegradable polyester composites [41]. The epoxy groups present in this chain extender can react with the hydroxyl and carbonyl groups of PBAT and with the hydroxyl groups of starch. One possible reaction pathway for the chain extender in PBAT/starch blends is the ring opening of the epoxy groups of ADR-4368, which can form an enhanced chain entanglement or crosslinking with both PBAT and starch, as shown in Figure 5 [42].

Lang et al. investigated the effects of Joncryl-ADR-4368 as a chain extender in PBAT/TPS composites with microcrystalline cellulose. They found that when using 2 wt% microcrystalline cellulose, the PBAT/TPS samples with the chain extender had about 80% higher elongation at break values than the samples without the chain extender, while the tensile strength was similar in both cases. This indicates that ADR-4368 mainly affects the ductility of the materials. In addition, the thermal stability and crystallinity of the samples were improved with the chain extender [41].

Wei et al. prepared three different types of modified PBAT using 2,2′-(1,3-phenylene)-bis(2-oxazoline) (PBO), poly(propylene glycol) diglycidyl ether (PPGDE), or Joncryl-ADR-4368 as chain extenders in PBAT/TPS blends. These chain extenders were used to increase the molecular weight of PBAT by a reactive extrusion process. Using 20 wt% of the modified PBAT with 40 wt% of regular PBAT and 40 wt% of TPS allowed for better dispersion of TPS, resulting in improved mechanical properties [43].

Marinho et al. prepared PBAT/TPS blends with different starch concentrations and with the addition of Joncryl PRO10, varying the processing temperature. Joncryl PRO10 is an epoxy oligomer with methacrylate residues recommended to compensate for degradation of polyesters during the processing and is able to extend the chain and establish compatibility between PBAT and TPS. The author observed that the chain extender was able to partially restore the chain integrity of PBAT, especially at higher processing temperatures (200 °C) and lower starch concentrations (10 wt%) [7].

Surendren et al. prepared PBAT and plasticized post-industrial wheat starch (PPWS) films with talc as filler and Joncryl-ADR-4368 to achieve exfoliation and high dispersion of talc. The chain extender was added at two extruder processing temperatures: 160 °C and 180 °C. The addition of talc alone drastically decreased the tensile strength and elongation at break values. On the other hand, the addition of the chain extender at 160 °C improved the tensile strength, and the elongation at break was more than twice that of the samples with talc alone. At 180 °C, the chain extender also improved the tensile strength, but the elongation at break was similar to the samples with only talc. These results indicate that the chain extender improves the interaction and interfacial adhesion of the fillers with the matrix [42].

Ju et al. prepared ternary TPS/PLA/PBAT blends with different concentrations of ADR-4468 as a chain extender with improvement in the tensile strength, elongation at break, and impact strength of the materials. From SEM image analysis, when the ADR-4468 concentration increased from 0 to 1%, there was no reduction on the agglomeration of starch particles in the blends. However, the compatibility between PLA and PBAT was improved by the addition of a chain extender, indicating that the chain extender reacted only with the PLA chain ends and the PBAT segments [41].

### 4.6. Compatibilizers/Additives

To achieve satisfactory physico-mechanical performance, the blend must have a favorable interfacial tension to limit the size of the dispersed phase, increase interfacial adhesion, and improve stress transfer between the phases [18]. The compatibilization strategy is usually a reactive extrusion process, as functional groups can be grafted into the polymer chains quickly and continuously [13]. To achieve this, a compatibilizer, which can be a graft or block copolymer, is added during the blending process or generated in situ. The degree of compatibilization of the blend depends on the reactivity of the compatibilizer. The effect of the compatibilizer can be attributed to two mechanisms: it reduces the interfacial tension between the phases and reduces the agglomeration of the domains through steric stabilization [18].

#### 4.6.1. Carboxylic Acids

**Figure 6:**
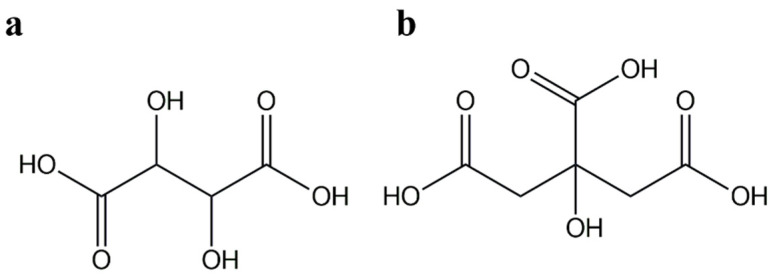
Molecular structure of (**a**) tartaric and (**b**) citric acid.

Natural based organic acids such as tartaric acid (TA) and citric acid (CA) (Figure 6) can interact with the hydroxyl groups of starch and introduce new carboxyl and ester groups into their structure [44]. The esterification of starch with carboxylic acids has been described as a method to produce less hydrophilic starch esters and to promote transesterification reactions (crosslinking) [16,44,45]. In terms of safety and health, natural organic acids have the advantage of being non-toxic and non-volatile, and they are already used as additives in food products [16,45].

##### Tartaric Acid

Tartaric acid is an organic dicarboxylic acid that acts as a compatibilizer through esterification and transesterification reactions with polymer chains restricting their mobility and introducing new potentially reactive carboxyl groups into the starch chains [16,44]. It can also promote hydrolysis of starch chains; and at high concentrations, this process can be excessive and reduce the tensile strength of the material [16].

Olivato et al. prepared PBAT/starch films using TA as a compatibilizer. It was found that the addition of TA promoted compatibility between the PBAT and TPS phases by reducing the interfacial tension, resulting in more homogeneous films. However, as the TA content increased, the opacity of the films increased [16].

Zhang et al. produced PBAT/TPS-TA composites. They found that TA and glycerol can migrate from TPS-TA to the PBAT matrix during extrusion with TPS-TA, which can act as a plasticizer for PBAT and consequently reduce the storage modulus (E’) of the composites. The SEM images showed that the addition of 0.5 to 2 wt% TA improved the compatibility between the PBAT matrix and the TPS-TA phase, but when 4 wt% TA was added, the composite showed poor compatibility and low interfacial adhesion [46].

Li et al. prepared PBAT/TPS films with TA by reactive extrusion. The rheological results of TPS showed that a higher TA content can compensate for a lower glycerol content by reducing the size of the TPS phase in the PBAT matrix and improving its dispersion. To adjust the glycerol and TA concentrations, the “lever principle” was applied, which takes into account the content and role of each component. Since 0.1 wt% TA (at 20 wt% glycerol) improves the mechanical properties of the material in a similar way to 2.5 wt% glycerol, the role of TA is 25 times greater than that of glycerol. This can be used to adjust the TA and glycerol content to reduce migration and improve hydrogen bond strength [47].

##### Citric Acid

The carboxylic groups present in the citric acid structure are polar groups that react with the hydroxyl group of starch and reduce its hydrophilicity, improving its compatibility with PBAT [13,32]. Depending on the interactions and reactions with the polymers in the blend, CA can act as compatibilizer, plasticizer, crosslinking agent or hydrolyzing agent [48].

Beluci et al. prepared PBS/PBAT/TPS films and evaluated the effect of CA on the properties of the films. It was found that using only 0.1 wt% of citric acid the water vapor permeability (WVP) of the films reduces in ~25%. However, there were no significant changes in the thermal stability of the materials [49].

Olivato et al. prepared starch/PBAT mixtures using glycerol as plasticizer and CA as a compatibilizer. The addition of CA led to a reduction in WVP values. Elongation at break was positively influenced by the interaction between glycerol and CA, indicating that CA can act as both a plasticizer and a crosslinking agent. However, at lower concentrations (0.75 wt%), the plasticizing effect is more pronounced. In addition, the increase in crosslinking reactions with 1.5 wt% CA also led to higher tensile strength values [50].

Garcia et al. studied the effects of using sodium hypophosphite (SHP) as a catalyst for the esterification of starch by CA in starch/PBAT films. The CA-containing films exhibited higher opacity due to the hydrolysis of the starch chains, which partially destroys the branched amylopectin structure and creates a more linear structure that increases the crystallinity of the material. The tensile strength and elongation at break values of the films with only CA were almost 1.5 and 2 times higher, respectively, than those of the control film (starch/PBAT). In these films, the addition of CA reduced the WVP of the films in 40%. However, the films prepared in the presence of SHP exhibited similar properties to the films containing only CA [51].

In contrast, Fourati et al. found that when 4 wt% CA was used as a compatibilizer in flat die extruded TPS/PBAT films with a weight ratio of 60/40, the mechanical properties of the film were severely affected. SEM analysis revealed that the 4 wt% CA resulted in a phase inversion where TPS was the continuous matrix and PBAT was the dispersed phase [6].

#### 4.6.2. Maleic Anhydride and Malleated PBAT

The free-radical grafting of maleic anhydride (MA) by reactive extrusion to incorporate pendant functional groups along the polymer backbone has received much attention in recent years. MA does not readily undergo homopolymerization under typical grafting conditions, allowing for a significant amount of MA to be incorporated into the polymer backbone. This results in higher grafting efficiency, as more MA is available for the intended reaction [18]. Previous studies have reported the compatibilizing effect in PBAT/TPS blends of using MA directly during the extrusion process or previous grafting MA onto the PBAT chain. When malleated PBAT (PBATg-MA) is used as a compatibilizing agent, the grafted MA reacts with the hydroxyl groups of starch and forms ester bonds that improve interfacial adhesion while controlling the size of the dispersed TPS phase [5].

Fourati et al. prepared thin films of TPS/PBAT blends by extrusion with different MA and PBATg-MA contents. It was found that when 2 wt% MA was used directly, the elongation at break of the film was reduced by more than 90% when compared to the control sample (TPS/PBAT). On the other hand, the elongation at break increased significantly with PBATg-MA at 2 wt%, reaching more than twice the value of the control samples. A gravimetric method was used to perform the continuity analysis of the different blends, and it was revealed that the blends with no compatibilizer had PBAT as its continuous phase, while the addition of 2 wt% MA in a mixture of TPS/PBAT (60/40) resulted in TPS being the continuous matrix and PBAT the dispersed phase. However, when PBATg-MA was used, the blend exhibited a co-continuous morphology, with both the TPS and PBAT phases contributing to the viscoelastic properties of the material, most likely due to enhanced interfacial adhesion caused by this compatibilizer [6].

Dammak et al. also investigated the influence of PBATg-MA and MA as compatibilizers on the mechanical properties, morphology, melt rheology, and biodegradability of PBAT/TPS blends with different proportions of TPS. The tensile strength and elongation at break were higher when PBATg-MA was used compared to MA. All blends proved to be biodegradable, but with different CO_2_ production rates. The biodegradation rates of the blends without compatibilizer were the highest, followed by the blends with MA, and lastly the blends with PBATg-MA [5].

**Figure 7:**
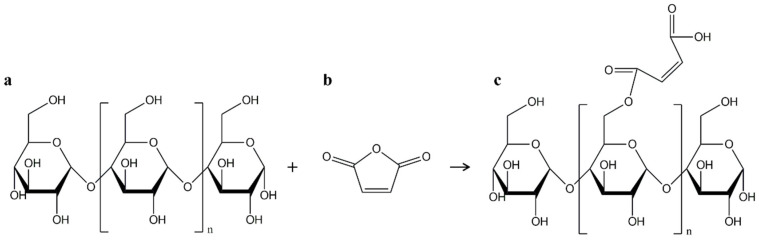
Starch esterification with MA. (**a**) Starch; (**b**) MA; and (**c**) starch ester (malleated starch). Adapted from [52].

MA has also been used to prepare starch esters with free carboxyl groups in starch structure (Figure 7) that are important for esterification with polyesters such as PBAT [18,52]. Raquez et al. prepared PBAT/malleated TPS (MTPS) films. It was found that the use of MTPS instead of MA (at the same MA content of 2.5 wt%) in blends with 70 wt% PBAT resulted in ~142 and ~40% higher values for tensile strength and elongation at break, respectively [52].

Chang et al. investigated the use of MTPS as a compatibilizer in multilayer PBAT/TPS films. Using MTPS with 2.5 phr MA based on TPS mass improved the chemical compatibilization between PBAT and TPS in blends with 30–50 wt% PBAT. The use of MTPS resulted in higher values of elongation at break, especially at 50 wt% MTPS, reaching a ~77% increase compared to the films prepared with TPS; however, the tensile strength of the films was similar for every formulation. Although films composed of only MTPS presented a 30% decrease in the WVP compared to TPS films, at final there were no significant differences in the WVP of the PBAT/MTPS films compared to PBAT/TPS [53].

#### 4.6.3. Soybean Oil

The addition of hydrophobic substances such as fatty acids and oils can reduce the hygroscopicity of PBAT/TPS films, which lowers the WVP and generally affects the tensile properties of the material. It has been shown that the addition of surfactants together with this class of substances can enhance the mechanical properties and WVP of the blended materials [54].

Soybean oil is a renewable and abundant raw material that contains various triglycerides in its chemical composition. The main acid components are oleic acid, linoleic acid, and linolenic acid. Brandelero et al. prepared TPS/PBAT films by adding soybean oil and Tween 80 (ethoxylated sorbitan monooleate), which have a high hydrophilic/lipophilic balance. The addition of soybean oil allowed more TPS to be incorporated without compromising the mechanical properties of the films. This can be explained by the compatibilization effect provided by soybean oil, which is related to the increase in groups that either interact to the ester groups of PBAT or to the glucose ring of starch, enhancing the interfacial adhesion between both polymers. The function of Tween 80 seems to be to promote the presence of glycerol between the starch chains, increasing their mobility; however, this effect was only observed when small amounts of soybean oil were used (0.5 wt% based on the starch mass) [54].

Epoxidized soybean oil (ESO) is produced by the epoxidation of soybean oil and is mainly used as a plasticizer for polyvinyl chloride (PVC) and chlorinated rubber. However, studies have shown that ESO can also be used as a plasticizer for starch and as a compatibilizer for polymer blends such as TPS and polylactic acid (PLA) [37]. Wang et al. prepared PBAT/TPS films, in which the TPS was previously prepared using glycerol and ESO as co-plasticizers, which helps to disrupt the crystalline structure of starch. The samples with only ESO as plasticizer (30 wt% based on starch mass) presented the better compatibilization between PBAT and TPS. But the higher tensile strength and elongation at break values were obtained when both plasticizers were used (20 wt% glycerol and 10 wt% ESO based on starch mass) [37].

Jiang et al. prepared PBAT/TPS complexes using ESO as a reactive compatibilizer. With only 5 wt% ESO, the compatibility between PBAT and TPS was greatly improved due to the chemical bond formed by ESO at the interface of the two polymers. Consequently, the mechanical properties of the compatibilized material were excellent. The transparency of the films was also improved by the addition of the same amount ESO [55].

#### 4.6.4. Cellulose

Cellulose is the most abundant biopolymer on earth. Its high crystallinity gives it high strength and stiffness. The similar chemical structure and polarity of cellulose and starch make cellulose an effective and compatible reinforcing agent for starch-based materials, resulting in improved mechanical properties while maintaining biodegradability. However, cellulose cannot be properly plasticized and dispersed, which hinders its application in PBAT/TPS films via extrusion. The high content of polyhydroxyl groups in natural cellulosic materials can aggregate through hydrogen bonding or van der Waals forces, which limits the reinforcing effect of cellulose on the films. Therefore, modifications or alternative forms of cellulose may be beneficial to improve dispersion and compatibility [56].

Microcrystalline cellulose (MCC) is one form of cellulose that is suitable as a reinforcing material for composites due to its excellent biodegradability, low cost, and high strength. While MCC does not shield hydroxyl groups, its smaller particle size and higher crystallinity can improve dispersion and reinforcement [41]. Lang et al. studied the effects of MCC in PBAT/TPS composites using a chain extender. The addition of MCC improved the mechanical and thermal properties of the blend, with optimal performance at 4 wt%. With a MCC content of more than 6 wt%, the mechanical and thermal properties of the composite deteriorated [41].

To address the issue of hydroxyl groups more directly, Jiang et al. prepared starch/PBAT films reinforced with two types of chemically modified celluloses: sodium carboxymethyl cellulose (CMC) and hydroxypropyl methyl cellulose (HPMC), and also with MCC. The films were prepared by conventional blown film extrusion using different addition methods: powder, aqueous solution, and emulsion. The films to which cellulose was added in the form of an emulsion showed better dispersion of the cellulose in the films. Among the different cellulose types, HPMC resulted in a uniform microstructure with better mechanical properties, surface hydrophobicity, water resistance, and barrier properties of the films, especially when added as an emulsion. This suggests that HPMC can effectively shield the hydroxyl groups and improve the compatibility between PBAT and TPS [56].

#### 4.6.5. Nanofillers

In recent years, there has been a particular interest in tailoring the structure and composition of materials by using materials with nanometer sizes. These nanofillers have at least one of their dimensions on the nanometer scale and have a high specific surface area that can significantly improve the properties of the polymer nanocomposite at low contents [18]. The advantage of using nanofillers is that only a low content may be required due to their excellent mechanical properties [57].

The use of nanofillers as reinforcing agents in the TPS phase of blends can reduce the water sensitivity and improve the mechanical properties without impairing the biodegradability of the material [57]. Hydrophilic fillers are most frequently reported in the literature, but there are also studies on hydrophobic nanofillers, such as nanoclays [58].

An important factor in the use of nanofillers is their dispersion, which affects the mechanical and barrier performance of the final nanocomposites that could result in a loss of reinforcing effect [59,60]. A very effective method to improve the dispersion of nanofillers in PBAT/TPS mixtures is high-energy ball milling, which uses mechanical force and the impact of grinding balls to complete uniformity of the powders [59].

##### Starch Nanoparticles

Starch nanoparticles (SNPs) are hydrophilic nanofillers that are highly compatible with PBAT/starch mixtures due to the large number of hydroxyl groups on their surface [58]. Normally, SNPs are prepared by acid hydrolysis. However, for industrial applications this synthetic method has a negative impact on the environment. Therefore, other methods such as gamma irradiation and ultrasound have been investigated as environmentally friendly methods for the production of SNPs [11,58].

Seligra et al. investigated the effects of gamma irradiation-produced SNPs on the compatibility of PBAT/TPS films. The incorporation of 0.6 wt% SNPs led to a slight improvement in the compatibility between PBAT and TPS, as evidenced by a reduction in the number and size of starch granules. In addition, the results of DMTA show that there was a shift in the starch-rich phase glass transition to lower temperatures, which is explained by the role of SNPs in the gelatinization of starch during extrusion, possibly contributing to a higher moisture content. In addition, both the elastic modulus and tensile strength of the blends were increased, while the elongation at break remained almost unchanged, demonstrating the reinforcing effect of SNPs in PBAT/TPS blends. Finally, the biodegradation of the films was accelerated with the addition of SNPs, which can be explained by the enhanced moisture content in compatibilized samples [58].

Silva et al. prepared PBAT/TPS films by thermoplastic extrusion with different amounts of SNP generated by ultrasound. It was reported that SNP had no effect on the morphology of the PBAT/TPS films, but as the SNP content increased, the relative crystallinity of the films decreased. TGA analysis showed that the addition of SNP led to a slightly higher decomposition temperature of the first degradation event, while DSC analysis revealed no change in the glass transition or melting temperatures of PBAT in the films when SNP was used. It was also found that the addition of SNP, even at 1 wt%, improved the elastic modulus in ~37% and the elongation at break in ~35%, and reduced in ~65% the WVP and in ~45% the water absorption [11].

##### Nanocellulose

Nanosized cellulose, either as cellulose nanofibers (CNFs) or cellulose nanocrystals/nanowhiskers (CNCs/CNWs), is a bio-based nanofiller that is frequently described in the literature as a reinforcing agent for various matrices [6]. When incorporated into films, nanocellulose improves the mechanical, barrier, and thermal properties of the material while maintaining its biodegradability [13]. In addition, CNFs can form chemical and physical bonds with starch chains, which can prevent starch retrogradation [59].

The incorporation of CNFs as TPS reinforcement on PBAT/TPS-CNFs blends was reported by Fourati et al. This reinforcement led to an improvement in the interfacial adhesion between the dispersed TPS phase and the PBAT matrix, resulting in more effective stress transfer between the phases and consequently improving the mechanical properties of the film [57].

Silva et al. investigated the compatibilization effect of CNWs in PBAT/TPS-CNW blends. The addition of CNWs resulted in greater compatibility between PBAT and TPS. However, the WVP and permeability of the films were not significantly affected [61].

CNF has low interfacial interaction with PBAT which hinders the reinforcing effect on PBAT/TPS composites [59]. To solve this problem, nanocellulose can be easily modified via different reactions such as esterification, oxidation, halogenation and etherification. Zhou et al. studied the effects of using hydrophobically modified nanofibrillated cellulose (HMNC, prepared using palm wax and sucrose fatty acid ester) in PBAT/TPS films. The films with 0.6 wt% HMNC presented tensile strength value ~70% higher and a water vapor transmission rate ~38% lower than the films without it [60].

Kong et al. introduced esterified CNFs into the PBAT/TPS films in order to better interact with PBAT, improving the interfacial adhesion of the composite material. In addition, the dispersion of the CNFs was improved by ball milling. The tensile strength of the films with 7 wt% CNF enhanced by 59% in comparison to the samples without CNFs. Comparing the films prepared with and without ball milling, there was a 30% increase in the tensile strength of the ones prepared with it. Also, the addition of CNF helped to reduce the crystallinity of the films and the size of PBAT crystallites [59].

##### Nanoclays and Nano-Silica

In recent years, polymer-layered silicate nanocomposites have shown improved thermal stability, mechanical strength and gas impermeability. These properties, which are achieved with a relatively low content of layered silicate (<5 wt%), are due to the interactions between the polymers’ chains and the clays, which can be represented as intercalation and/or exfoliation/delamination structures [18]. PBAT/TPS blends can be reinforced with nanoclays, which are readily available, versatile, and environmentally friendly [62]. Montmorillonite, sepiolite, and bentonite are mineral clays that are widely used as nanoscale reinforcements for polymer composites [63].

Olivato et al. prepared PBAT/TPS blends with sepiolite nanoclay as reinforcement. Previous studies reported that sepiolite is mainly localized in the TPS-rich phase, which explains the greater improvement in the tensile strength of TPS/PBAT nano-biocomposites with higher TPS contents (80 wt%). These results are typical of good dispersion of nanofillers in nanocomposites [64].

Blends of PBAT/TPS with two different layered silicates as reinforcement, bentonite or organically modified montmorillonite (OMMT), were studied by Lendvai et al. The addition of 5 phr bentonite to TPS promoted a compatibilizing effect, resulting in higher elongation at break values, reaching 411.2% at 40 wt% TPS; however, the addition of OMMT had no significant effect on the tensile properties of the PBAT/TPS blends [63].

Dang et al. prepared PBAT/TPS blends reinforced with halloysite clay nanotubes (HNTs). Compared to other silicates, HNTs have a lower amount of hydroxyl groups on the surface, making them suitable as a reinforcing filler for polymer composites. Different PBAT/TPS ratios and HNT concentrations were tested, and the formulation with the higher tensile strength and elastic modulus was the one with an 80/20 PBAT/TPS ratio and 5 wt% HNT. This confirms the reinforcing effect of HNT and demonstrates its higher affinity and dispersion in the TPS phase, as shown by SEM images [62].

Nayak et al. prepared PBAT/TPS blends with two types of organically modified nanoclays (Cloisite 20A and Cloisite 30B). PBAT was grafted with MA through a reactive extrusion process that required a free radical initiator to induce its reaction with the hydroxyl groups of the starch and the nanoclays. With 3 wt% of nanoclays, the tensile properties of the PBAT matrix were enhanced, especially when Cloisite 30B was used. This can be explained by the more hydrophilic nature of the functional groups (quaternary ammonium salt) used to modify Cloisite 30B, which enabled a higher interaction with the TPS phase, enhancing its reinforcing effect [14].

**Figure 8:**
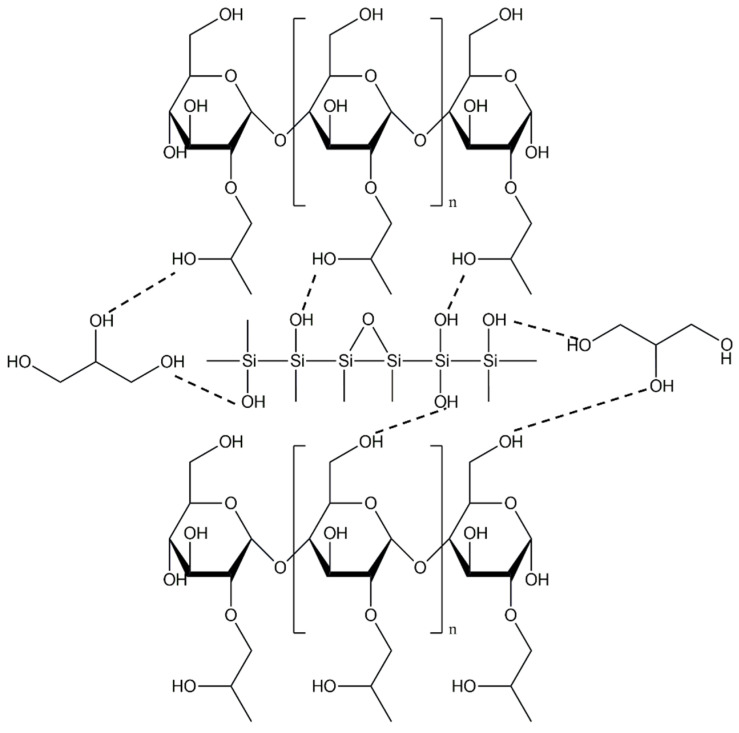
Interactions between hydroxypropylated starch, glycerol and nano-silica (dashed lines correspond to hydrogen bonds). Adapted from [65].

It is hypothesized that adding nano-silica to TPS may allow the incorporation of larger amounts in PBAT/TPS blends while maintaining the usability of the materials and also reduce the cost of the final blend [65]. Li et al. studied the mechanical properties of PBAT/TPS films with nano-silica incorporated into TPS made from hydroxypropylated starch. With 4 phr nano-silica and 20 wt% TPS, the tensile strength of the films reached a maximum of 13.5 MPa. The improvement in mechanical strength can be explained by the formation of hydrogen bonds between the hydroxyl groups of nano-silica and the large number of hydroxyl groups in the starch matrix (Figure 8). In addition, the addition of nano-silica allows the use of a higher TPS content (up to 40 wt%) in the blends while maintaining a high mechanical strength of the film [65].

#### 4.6.6. Talc

The use of talc can improve the physical properties of polymer composites due to the synergistic effect between the components of the matrix and the filler [66]. Talc is a hydrophobic filler that presents a layered structure of nanometric thickness and microsized length. It is an ideal filler for polymer composites due to its lubricity, exceptional wettability, and dispersion [42]. Surendren et al. prepared PBAT/PPWS films with 25 wt% talc as filler and Joncryl-ADR-4368 as chain extender to achieve talc exfoliation and higher degree of dispersion. The addition of talc resulted in a modulus of elasticity of the composite that was about 334% higher. However, the values for elongation at break and tensile strength were approximately 97 and 34% lower, respectively, which can be explained by the formation of stress concentration and crack initiation [42].

#### 4.6.7. Lignin

**Figure 9:**
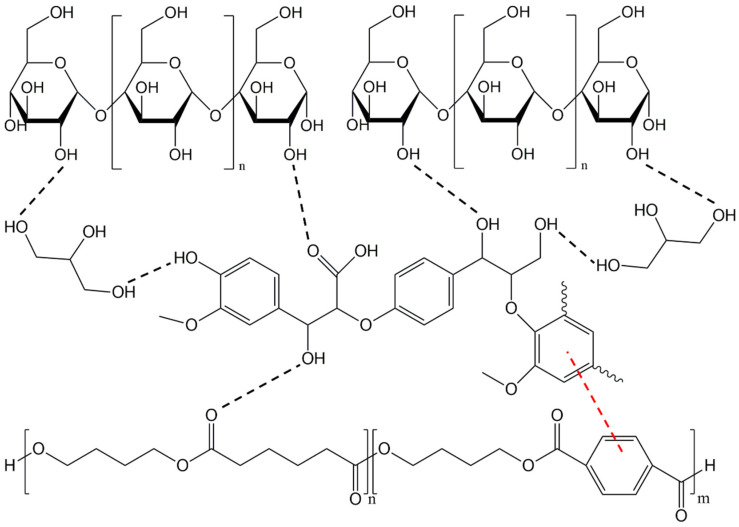
Interactions between starch, glycerol, lignin, and PBAT (black dashed lines correspond to hydrogen bonds and the red dashed line corresponds to π-electron interactions). Adapted from [67].

Lignin, which is found in wood, plants, and agricultural products, is the second largest type of biomass in the world. It is generally considered to be the only natural aromatic polymer with glass transition and is a suitable filler for various thermoplastic polymers. Due to its amphiphilic properties and intermolecular interactions, either through hydrogen bonds or *π*-electron interactions, lignin is a good candidate to act as a compatibilizer between PBAT and TPS (Figure 9) [67,68].

Li et al. prepared PBAT/TPS blends with lignin to investigate its effects on the interfacial compatibility between the two polymers. With 10 wt% lignin and 30 wt% TPS, the PBAT/TPS composites achieved maximum tensile strength (13.1 MPa) and elongation at break (917.1%). In addition, lignin improves the compatibility, thermal stability, and hydrophobicity of the blends [67].

Lignin also promotes UV protection due to the presence of an aromatic ring and a conjugated quinone structure. For materials that are directly exposed to sunlight such as mulch films, the UV-protective property is crucial, because it accelerates their aging and consequently leads to performance degradation and shorter lifetime [68]. Lin et al. introduced lignin into PBAT/modified starch films (with polyurethane prepolymer) to study their UV aging resistance. The addition of lignin improved the tensile strength and elongation at break of the films, with the values being higher at only 1 wt% lignin. The addition of lignin resulted in a lower performance degradation after UV aging. Lignin also enabled improved heat seal strength of the films [68].

#### 4.6.8. Polyurethane Prepolymer

**Figure 10:**
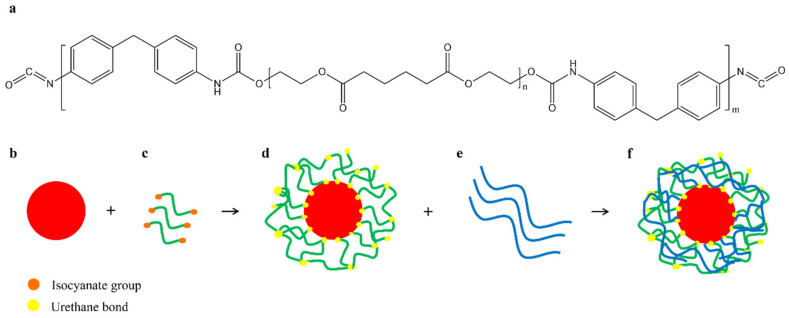
(**a**) Molecular structure of PUP and schematic representation of modification of starch with PUP and reactive blending with PBAT, where (**b**) is starch; (**c**) is PUP; (**d**) is modified starch; (**e**) is PBAT; and (**f**) is starch/PBAT composite. Adapted from [69].

Another way to improve the compatibility between PBAT and starch is to use a polyurethane prepolymer (PUP) to modify the starch. The isocyanate groups contained in PUP react with the hydroxyl groups of the starch and form a urethane bond, so that the starch granules are firmly coated by the PUP layers [25,68,69]. The PUP layers formed on the starch surface provide good compatibility with PBAT, and the aliphatic segments of PBAT interact with the soft segments of PUP [25,68,69]. A schematic representation of the reactive blending is shown in Figure 10.

Meng et al. prepared blends of a higher molecular weight PBAT and PUP-modified starch (MS). The elongation at break and tensile strength of the MPBAT/MS (50/50) blends increased from 3.8 MPa and 93.3% to 11.9 MPa and 580.4% with a PUP concentration in MS of 13 wt%. In addition, the thermostability and hydrophobicity of the composites increased with increasing PUP content [69].

#### 4.6.9. Sodium Nitrite

Sodium nitrite (SN) is a preservative that complies with international standards and regulations and is used to stabilize the color of meat products. In addition, SN is known as a starch plasticizer that also improves the homogeneity and compatibility of polymer blends. Tuntiworadet et al. prepared PBAT/TPS films with SN and studied its effects on the blend properties. As the SN content increased, the compatibility between PBAT and TPS improved and consequently the mechanical and oxygen barrier properties of the films also improved, reaching optimum performance at 1 wt% of SN. However, the thermal stability and WVP were negatively affected by the addition of SN due to the reduction of the molecular weight of starch and PBAT as a result of hydrolytic processes catalyzed by the acids formed from SN during extrusion [70].

#### 4.6.10. Polydimethylsiloxane

Polydimethylsiloxane (PDMS) consists of a siloxane backbone and methyl groups side chains. It is a polymer biocompatible, resistant to corrosion, thermally and oxidatively stable, hydrophobic, cost-effective, and with excellent film-forming capacity. PDMS has been used to produce superhydrophobic coatings for packaging. However, little information is available on the use of this polymer in direct addition and extrusion. Song et al. studied the effects of incorporating PDMS in PBAT/starch films. The addition of PDMS resulted in greater compatibility between PBAT and starch, leading to enhanced mechanical properties. With 2 wt% PDMS, the tensile strength and the elongation at break increased by 25% and 21%, and the water contact angle increased from 69 to 87°. Furthermore, the PBAT/starch films with 3 wt% of PDMS were tested as banana and mushroom packages, and they helped to extend the shelf life of these products due to their better barrier properties [71].

### 4.7. Ternary Blends

In addition to the compatibilizers, work is also being done on ternary blends, i.e., blends including PBAT, TPS, and another polymer with properties suitable for the desired application. The addition of this third polymer could create a synergistic effect and improve the properties of the blends.

#### 4.7.1. PBAT/TPS/PLA

The addition of poly(lactic acid) (PLA), an aliphatic hydrophobic polyester, to PBAT/TPS blends is interesting because it is not only biodegradable but also comes from a renewable source. PLA has similar properties to some non-biodegradable plastics such as polypropylene and poly(ethylene terephthalate). However, its brittleness and hydrolytic properties hinder its wide application. Therefore, blending PLA with PBAT and TPS can result in materials with good integrated properties [17].

Shirai et al. investigated the effects of the addition of PLA on the functional properties of TPS/PBAT films. It was found that the PBAT/TPS/PLA films had higher elastic modulus and tensile strength values and lower elongation at break and WVP than the PBAT/TPS films. The addition of PLA also significantly reduced the opacity of the films, due to the transparency and semi-crystallinity of PLA. It was concluded that none of the PBAT/TPS/PLA (40/50-30/10-30) films were suitable for use in flexible packaging due to their mechanical properties and thickness [17].

Zhao et al. investigated the quality of PBAT/PLA/TPS films with different TPS content (10–40 wt%) as packaging material for straw mushrooms. As expected, the tensile strength and elongation at break values gradually decreased with increasing TPS content (the PBAT:PLA ratio remained the same value of 4:1). In contrast, the films with 30 wt% TPS (56 wt% PBAT and 14 wt% PLA) presented the better results for the preservation of straw mushrooms [72].

#### 4.7.2. PBAT/TPS/PBS

Poly(butylene succinate) (PBS), like PBAT, is an aliphatic polyester that has suitable mechanical properties to replace non-biodegradable plastics; however, its wider use is hampered by its high cost. The films composed of PBS and PBAT exhibited high strength and flexibility [48,49].

Beluci et al. investigated the effects of different polymer ratios on PBAT/PBS/TPS films using citric acid as a compatibilizer. It was found that the films consisting of only TPS (with a fixed content of 70 wt%) and one of the polyesters, either PBS or PBAT, had inferior mechanical properties than the films containing TPS and both polyesters. While PBAT provided higher ductility, PBS contributed to higher strength, and TPS contributed to improve biodegradability [48].

The same authors investigated further the ternary blends of PBAT/PBS/TPS, focusing on the physical, thermal, and barrier properties of the films. The films with TPS (fixed at 70 wt%) and only one of the polyesters exhibited poorer barrier and thermal properties than the films from the ternary blend. With 10 wt% PBS and 20 wt% PBAT, the WVP of the films lowered 30% in comparison with the films with only TPS/PBS or TPS/PBAT [49].

#### 4.7.3. PBAT/TPS/Cellulose Acetate

Cellulose acetate is a cellulose derivative ester that can become thermoplastic when a plasticizer is added. Because the structures of starch and cellulose acetate are similar, there is great potential to produce blends of both polymers [73]. Moraes et al. prepared blends of TPS, plasticized cellulose acetate (PCA—Tenite™ acetate from Eastman Chemicals Co., Kingsport, TN, USA) and PBAT with different compositions and processing temperatures. The morphology and thermal properties of the PBAT/TPS/PCA films revealed a heterogeneous structure when PBAT was added to the TPS/PCA blend, which was explained by poor compatibilization between PCA and PBAT. When 50 wt% TPS, 25 wt% PCA, and 25 wt% PBAT were used, the tensile strength and elongation of the films were 74 and 89% lower, respectively, than the films with only TPS/PCA (50/50) [73].

### 4.8. Use of Additives for Active Packaging

Packaging is one of the most important factors for transportation, storage, and quality of food during its shelf life [13]. Traditionally, the choice of packaging material has been based solely on the ability of the material to protect the food it contains in a passive way. Recently, there has been increasing interest in the development of active packaging that can minimize the growth of microorganisms and oxidative reactions. Active packaging can absorb or release substances from the food inside the package or from the surrounding environment. Consequently, these features would extend the shelf life of the products and maintain their quality [10,13,61].

The main active packaging reported in the literature are systems that release substances, such as carbon dioxide, antioxidants, and antimicrobial compounds, and scavenging systems, such as ethylene absorbers, oxygen, and moisture scavengers [45]. Active packaging is an interesting alternative to the direct application of antioxidants or antimicrobial agents to food, as it can lead to a change in some sensory properties such as taste and appearance of the food [33].

In recent years, several studies have been conducted on active PBAT/TPS packaging with various antimicrobial, antifungal, and/or antioxidant agents such as sodium benzoate, potassium sorbate, nisin, EDTA, and nitrite [74,75,76]. Although artificial preservatives have high stability, economy, and efficiency, they are potentially harmful to health, which hinders their use [77]. Therefore, natural extracts such as essential oils and curcumin have recently gained attention due to their antimicrobial, antioxidant, and other biological properties [13].

#### 4.8.1. Essential Oils

Natural products from aromatic plants have attracted the attention of several researchers. Essential oils (EOs) are natural antibacterial and antioxidant compounds [78]. Essential oils are lipophilic and volatile compounds that are generally recognized as safe (GRAS). They are often used in active packaging for their antioxidant, antifungal, and antimicrobial activities [10].

Cinnamon oil has antibacterial, antioxidant, and anticarcinogenic properties that make it an interesting material for use in cosmetics, food, and medical applications, including active packaging [9,10]. Tian et al. developed PBAT/TPS films with cinnamon oil and investigated the effects on the properties of the films by varying the TPS content. It was found that as the TPS content increased, the retention of cinnamon oil during film preparation increased, which is explained by the hydrogen bonds formed between cinnamaldehyde and TPS. Consequently, the final films with higher TPS content contained more cinnamon oil, leading to a higher release of the oil from the film into the packaged food and consequently to a higher inhibition rate against *E. coli*, *S. aureus*, and *S. putrefaciens*. However, as expected, with increasing TPS content, the mechanical and WVP properties of the films decreased, while the oxygen barrier properties increased [10].

Peppermint and clove oils are suitable alternatives to synthetic antibacterial and antioxidant agents for food packaging [78]. Gui et al. investigated the effects of peppermint oil and clove oil in PBAT/TPS/PLA films on the preservation quality of straw mushrooms. The use of 5 wt% EOs led to a significant decrease in the tensile strength and elongation at break as well as the gas barrier properties of the films. However, there was a significant improvement in the antioxidant activity of the films. The films also inhibited straw mushroom autolysis, extending their shelf life, especially in films prepared with peppermint oil [78].

The addition of EOs in the form of nano-emulsions can improve the mechanical properties and antibacterial activity of composite materials. However, it is difficult to maintain the stability of nano-emulsions during processing and storage. To solve this problem, nano-emulsions can be processed into microcapsules by spray drying [9]. Hu et al. prepared PBAT/TPS films incorporated with cinnamon oil microcapsules (which also contained hydroxypropyl-β-cyclodextrin, ethyl lauroyl arginate, and maltodextrin) to improve antibacterial properties. The incorporation of microcapsules significantly increased the antibacterial activity against *E. coli* and *S. aureus*. In addition, the mechanical properties of the films with microcapsules were slightly better and the WVP was lower. The films were tested for packaging strawberries, and the addition of microcapsules was effective in extending the shelf life of the fruit while maintaining a good appearance [9].

A way to avoid the loss of EOs during processing, owing to their volatility, is to use crosslinked porous starch as a carrier for these substances. Gui et al. prepared PBAT/PLA/TPS films using three different methods to incorporate clove essential oil: direct loading, porous starch loading, and sodium trimetaphosphate-crosslinked porous starch (ScPS) loading. The films loaded using porous starch and ScPS exhibited poorer tensile strength and elongation at break values, which may be due to their porous structures. Comparing both, the films with the ScPS presented higher tensile strength, probably due to its denser structure and increased thermal stability provided by the crosslinking. In addition, the films loaded using ScPS presented good, sustained release of clove oil and were able to extend the shelf life of salmon by delaying microbial growth, lipid oxidation, and protein hydrolysis [79].

#### 4.8.2. Curcumin

Curcumin, extracted from turmeric, has been widely studied for its biological properties such as its antimicrobial and antioxidant activities [33,45]. Campos et al. investigated the antioxidant/antimicrobial effect of curcumin in TPS/PBAT films. The antioxidant capacity of the films increased 10 and 14 times when 0.5 wt% and 0.75 wt% of curcumin was incorporated. The films also exhibited satisfactory antibacterial activity against *E. coli* and *S. aureus*. The mechanical properties were not affected by the addition of 0.5 wt%, but when the content was increased to 0.75 wt%, the values for tensile strength and elongation at break values increased ~51% and ~43%, respectively. In contrast, 0.75 wt% curcumin resulted in slightly poorer thermal stability [33].

#### 4.8.3. Quercetin

Quercetin, 3,3′,4′,5,7-pentahydroxyflavone, is a polyphenolic molecule found in many vegetables and fruits. This compound has been shown to have antibacterial, antioxidant, anticancer and anti-UV activity. Yang et al. incorporated quercetin into PBAT/TPS films and added organically modified montmorillonite to improve the gas barrier properties and slow down the release rate of quercetin from the film, thereby prolonging the antioxidant activity. The addition of 1 phr quercetin and 15 phr OMMT increased in 36.2% the tensile strength and 35.7% the tear strength of the films, which is due to the formation of hydrogen bonds between the silanol groups in OMMT and the starch. Food preservation was tested with bananas and blueberries, and it was found that these fruits were better preserved when using the PBAT/TPS/quercetin/OMMT film (1 phr of quercetin and 15 phr of OMMT) compared to conventional LDPE films [80].

#### 4.8.4. Phenolic Compounds

Phenolic compounds, that can be extracted from plants such as rosemary and green tea, are promising antioxidants and antibacterial agents [77,80]. Zhai et al. investigated the effects of incorporating tea polyphenols (TPs) into PBAT/TPS films for efficient active packaging materials. The incorporation of TPs increased both antioxidant and antibacterial activity, and efficiency increased with increasing content. The mechanical properties decreased slightly with increasing the amount of TPs, which was explained by the agglomeration of the additives in the PBAT matrix at higher concentrations. However, the formation of hydrogen bonds between the phenolic groups and the starch chains prevented a significant deterioration of the mechanical properties. However, the barrier properties improved with increasing TP content. Furthermore, the addition of TP slowed down the short-term degradation but accelerated the long-term degradation of the films [77].

#### 4.8.5. Oxidized Starch

Oxidized starch (OST) can be produced by reacting starch with oxidizing agents such as hydrogen peroxide, under certain conditions. The oxidation of some of the hydroxyl groups of starch results in a high content of carboxyl groups, which creates an environment that hinders bacterial survival. Since some bioactive substances impair the mechanical properties of the films, OST could be a promising alternative for the food packaging industry, providing antimicrobial properties without compromising the mechanical properties [81]. Li et al. prepared OST/PBAT films to investigate the suitability of OST as an antibacterial agent. The antimicrobial activity increased with an increasing OST content, reaching an inhibition rate of 98.9% (against *S. aureus*) at 40 wt%. The addition of OST led to a slight deterioration in the mechanical properties of the films; however, even blends with 40 wt% OST still achieved the minimum tensile strength value required by the GB10457-2021 (China) directive for food packaging applications [82]. In addition, the OST/PBAT films (40/60) were able to extend the shelf life of fresh pork at 4 °C by 2 days compared to PE films [81].

#### 4.8.6. Oxide Nanomaterials

Several oxide nanomaterials such as copper oxide nanoparticles (CuONPs), zinc oxide nanoparticles (ZnONPs). and titanium dioxide nanoparticles (TiO_2_NPs) have antimicrobial properties that make them suitable candidates for use in active packaging applications. In addition, the hydrogen bonds formed between the metal oxide nanoparticles and polymeric materials reduce the hydrophilic sites in the polymer chain, leading to lower water absorption and the water sensitivity of the composites [83,84,85]. Nevertheless, there are concerns about the migration of nanomaterials from packaging materials into food, which may affect consumer safety and the organoleptic properties of food. This can occur through the diffusion-based release of solubilized particles and ions, or the release of particles can occur through the abrasion of the packaging surface [85]. The release of nanoparticles from the packaging under unfavorable conditions can lead to the permissible migration threshold in food being exceeded, which changes the film properties and reduces the effectiveness of the antibacterial activity [8]. Therefore, determining the potential migration behavior of the packaging is crucial for its commercial use.

Due to their thermal stability, comparatively lower toxicity, high surface-to-volume ratio and improved mechanical properties, CuONPs have been widely used to improve the properties of polymer films as antioxidant and antimicrobial agents in active packaging [83]. Bumbudsanpharoke et al. prepared PBAT/TPS films with CuONPs for use in active packaging. With 2 wt% CuONPs, the seal strength of the films was ~26% higher than the control samples, which can be explained by the increased thermal energy diffusion through the polymer leading to easier melting during the heat-sealing process, and consequently a higher interfacial interaction between the contact surfaces and a stronger sealed film. The tensile strength and elongation at break of the films were also enhanced by the addition of CuONPs, which is due to the hydrogen bonds formed between the metal oxide and the TPS matrix. The incorporation of CuONPs improved the gas barrier properties of the film, which showed over 99% of reduction in the *E. coli* colony count, demonstrating an effective antimicrobial activity [83].

ZnONPs are characterized by high stability, antimicrobial activity, low cost, UV-blocking properties, and low toxicity [8,84,85]. Phothisarattana et al. prepared PBAT/TPS films with ZnONPs for active packaging applications. Increasing the ZnONP content to 3 wt% (based on starch weight) resulted in agglomeration of the nanoparticles which decreased the compatibility between PBAT and TPS and resulted in poorer mechanical and barrier properties. However, the films were very efficient in preventing microbial growth and lipid oxidation when used in meat packaging [84].

Phothisarattana et al. studied the effects of nanoparticles of titanium oxide (TiO_2_NPs) and zinc oxide (ZnONPs) in PBAT/TPS films, particularly with regard to their migration mechanisms. TiO_2_NP is commonly used in the cosmetic and food industries as a relatively inexpensive and stable nanofiller with strong antimicrobial activity and ethylene scavenging activity. It was found that TiO_2_NPs promoted higher water absorption than ZnONPs, leading to plasticization effects that facilitate granular breakdown of the starch phase. The migration behavior of the films depended on the type of simulant used, and it was concluded that the films can be used in contact with water and acidic foods (with the exception of films containing more than 4 wt% ZnONPs, which should not be used in contact with acidic foods) [85].

Bumbudsanpharoke et al. investigated the stability of ZnONPs in PBAT/TPS films after contact with food simulants. When ethanol was used as a food simulant, all films showed migration values below the specified limit of 5 mg/kg food (Commission Regulation (EU) 2016/1416). However, under acidic conditions and at 1 wt% ZnONP, the migration value exceeded the specified limit. This can be explained by the higher solubility of ZnO in an acidic environment, which leads to its dissolution. In terms of mechanical properties, ZnONP at 3 wt%. acted as a reinforcing agent in the films and it achieved an optimum mechanical performance. The films also exhibited good UV blocking properties and a high inhibition rate against *E. coli*, reaching values above 99.9% for all formulations [8].

#### 4.8.7. Sodium Benzoate and Potassium Sorbate

Sodium benzoate (SB) and potassium sorbate (PS) are food preservatives classified as GRAS, which are usually applied to starch-based foods to extend their shelf life [76]. Wangprasertkul et al. studied the effects of incorporating SB and PS into PBAT/TPS films. PS is a linear alkene hydrocarbon, which easily disperses in the PBAT and TPS networks, and its carboxyl groups interact with starch, resulting in stronger compatibility between the two polymers. On the other hand, the bulky benzene rings of SB promoted poorer dispersion in the PBAT and TPS networks. The addition of 6 wt% PS to a PBAT/TPS blend (60/40) led to an increase in the tensile strength and elongation at break of the films, while the addition of BS at any content worsened the mechanical properties. Films made with 6 wt% SB, 6 wt% PS, or 6 wt% SB/PS (with a 1:1 ratio) were all effective in retarding microbial growth and inhibiting mold growth (up to 8 days) in rice noodles [76].

#### 4.8.8. Quaternary Ammonium Salts

Quaternary ammonium salts (QAS) have been used in antiseptics and disinfectants in recent decades due to their excellent antimicrobial activity, low cost, and low toxicity [86]. Gao et al. incorporated commercially used QAS as didodecyl dimethyl ammonium chloride (D1221) and dioctadecyl dimethyl ammonium chloride (D1821) into PBAT/starch films. The longer chains of D1821 improved the miscibility and consequently the compatibility between PBAT and starch. At 1 wt% D1821, the tensile strength and elongation at break of the films increased, which can be explained by the improved interfacial adhesion between the polymers. However, at higher levels of D1821 or at any level of D1221, all mechanical properties deteriorated, probably due to the formation of aggregates of the hydrophobic portion of the salts between the starch chains. In contrast, D1221 showed significantly higher antibacterial activity against *S. aureus* and *E. coli*, especially at 5 wt%. In addition, D1221 was also the most promising preservative in meat packaging at 5 wt% [86].

#### 4.8.9. Propyl Gallate

The addition of natural antioxidants can have disadvantages such as high cost, poor thermal stability, and bad odor. Therefore, the use of synthetic commercial antioxidants such as propyl gallate (PG) is a stable and cost-effective alternative. In addition, they have a good safety record within the approved ingredients [87]. Gao et al. prepared PBAT/starch films as vehicles for PG, hypothesizing that starch could be a smart gatekeeper for controlling PG release. It was found that sustained PG release could be controlled by adjusting the PBAT/starch ratio and that higher starch amounts led to increased swelling degrees. When ethanol solutions were used as food simulants, especially at high concentrations, there was a rapid release and large migration of PG. PBAT/starch films with 3 wt% PG presented a great antioxidant activity when used as peanut butter packages. These films are promising as active packaging materials for lipid-rich products [87].

#### 4.8.10. Polyhexamethylene Guanidine Hydrochloride

**Figure 11:**
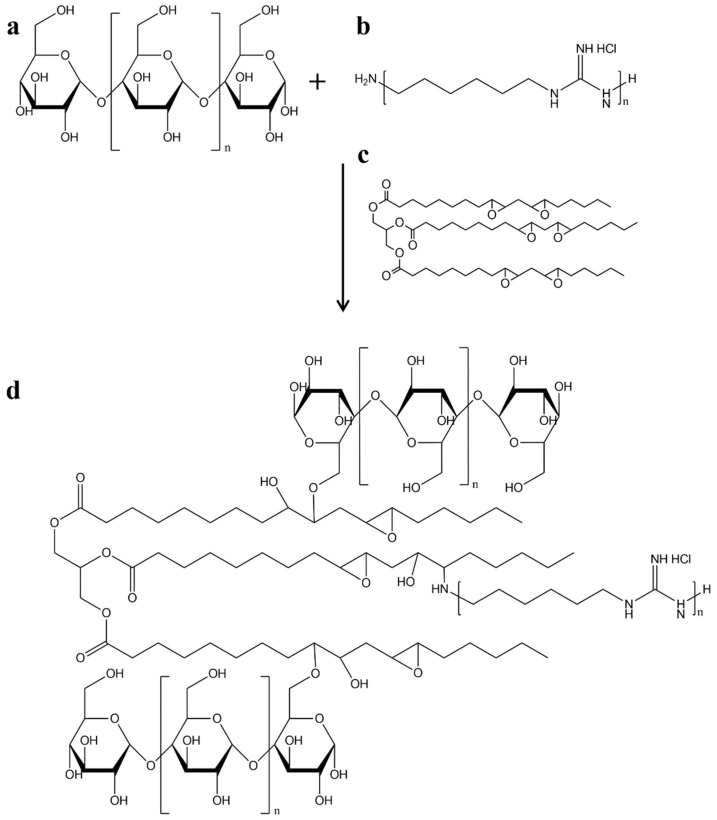
Reaction between (**a**) starch, (**b**) PHMG, and (**c**) ESO via reactive melt extrusion, resulting in (**d**) modified starch with ESO grafted with PHMG. Adapted from [88].

Polyhexamethylene guanidine hydrochloride (PHMG), an excellent broad spectrum antibacterial agent, is chemically stable, relatively non-toxic, minimally harmful to the environment, and inexpensive. However, direct mixing with polymers often leads to fast leaching due to the high solubility of PHMG in polar solvents. One solution is therefore to graft PHMG onto carrier polymers, such as epoxidized soybean oil, which increases the coupling efficiency and can synergistically improve the mechanical and antibacterial properties due to the large number of epoxy groups [88]. Liu et al. prepared PBAT/TPS films using ESO as crosslinking agent and PHMG as an antibacterial agent (Figure 11). During melt extrusion, the epoxy groups present in TPS-E (TPS with ESO and PHMG) possibly reacted with PBAT, resulting in the formation of a micro-crosslinking network structure. This structure is responsible for the increase in the tensile strength and elastic modulus of all films to which PHMG and ESO were added. At a PBAT/TPS ratio of 70/30, the addition of 5 wt% ESO and 5 wt% PHMG (based on starch weight) resulted in films with tensile strength and elastic modulus 112 and 52.4% higher, respectively, while the elongation at break slightly decreased. The films exhibited no leaching of PHMG and showed excellent antibacterial activity [88].

#### 4.8.11. Blueberry Extract

Microbial growth and fluctuations in metabolite content due to food spoilage led to pH variations. Therefore, colorimetric pH-responsive films are promising for real-time monitoring of food quality. Anthocyanins, one of the class of polyphenols present in of blueberry extract (BE), show a colorimetric pH response [89]. Gao et al. prepared PBAT/starch films with BE to achieve pH-responsive packaging. With 2 wt% BE, the tensile strength and elongation at break of the films reached a maximum value of 7.85 MPa and 606.53%, with an increase of ~21% and ~23%, respectively, compared to the control samples. The films also exhibited high UV resistance and good gas barrier properties. The films were used as shrimp packaging, indicating great potential for the production of biodegradable, pH-responsive packaging without excessive leaching of pigments [89].

### 4.9. Foams

Commercially available foam plastic packaging is non-biodegradable, light, and bulky, which leads to disposal problems. In addition, there are no economically and ecologically viable recycling processes due to the high handling and transportation costs. There is therefore a need for biodegradable foam packaging that meets the requirements for this application [90].

Starches with high amylose content provide a structural platform for the production of biodegradable foams, and many studies have reported starch-based foams with additives to reinforce TPS using water as a foaming agent [90,91]. However, the hydrophilicity and thermal sensitivity of starch hinder its wider application. To overcome this problem and maintain the biodegradability of the foam, researchers have reported melt blends of TPS with other hydrophobic biodegradable polymers, including PBAT [90].

Nabar et al. prepared starch foams in the presence of PBAT and used PBATg-MA as a compatibilizer. Water was used as plasticizer and foaming agent, and talc as nucleating agent. PBATg-MA proved to be an efficient compatibilizer that provides maximum resiliency and minimum density to the starch foams. The lowest density (21.2 kg/m^3^) was achieved at 0.5 wt% PBATg-MA and 4.5 wt% PBAT. In addition, the starch foams with PBAT and PBATg-MA showed improved hydrophobic properties, i.e., they absorbed less weight in the presence of moisture [90].

Chang et al. prepared TPS/PBAT blends with silane A (SA) as a compatibilizer and foamed them with supercritical CO_2_ at different foaming temperatures and pressures. The SA has three methoxy groups and one epoxy group, and can be used as a functional group modifier and coupling agent to modify the terminal functional groups of TPS. By extending the TPS/PBAT molecular chains and creating intermolecular entanglements between the polymers, the resulting composites exhibited elasticity and buffering properties that can support cell structure during foaming process. It was found that the TPS/PBAT composite was not suitable for the production of foams due to poor interfacial adhesion between the polymers. However, when SA was added, the foam exhibited uniform bubbles with a closed cell structure, resulting in lower foam density and better tensile properties. The lowest foam density (160 kg/m^3^) was obtained for the samples with 50 wt% PBAT and 50 wt% SA/TPS (10 phr SA) processed at 100 °C. Increasing the SA/TPS concentration (60–80 wt%) was unfavorable for the composites as higher temperatures were required to achieve the lowest densities. Even then, the lowest densities were significantly higher than those of the samples with 50 wt% SA/TPS [91].

The same research group investigated PEG-modified TPS/PBAT foams with similar conditions at different foaming temperatures and CO_2_ pressures. TPS was the main constituent of the blend. PEG was used as a plasticizer to increase structural melt strength, originating foams with better elasticity and buffering properties. Different molecular weights of PEG (1000, 2000, and 3000) were tested, and SA was used as a compatibilizer between PEG-modified TPS and PBAT. The blends incorporating the lowest molecular weight PEG showed the lowest foam density in all cases, with or without SA. In other cases, the samples without SA generally produce foam with a lower density. The lowest foam density was achieved at a foaming temperature of 100 °C and a foaming pressure of 17 MPa. The tensile properties of the foams were enhanced by the addition of PEG. The addition of SA led to significantly higher tensile strength values; however, the elongation at break was not significantly changed [92].

### 4.10. 3D Printing

Research in the field of additive manufacturing has increased in recent years due to the flexible customization of products and the ability to use different materials in the printing process. One of the most popular additive manufacturing methods is fused deposition modelling (FDM), as it is relatively inexpensive and easy to handle. In this process, thermoplastic materials are used to produce 3D models by melting the raw materials at high temperatures, extruding them through a nozzle and building them up layer by layer until the model is finished [40].

Due to environmental concerns, there is an increasing search for biodegradable materials for 3D printing. Ju et al. produced ternary TPS/PLA/PBAT blends for use as filaments in FDM 3D printing applications. Using 1 wt% of a chain extender, the 3D printed samples had almost no gaps at the interface suture of the blends, resulting in good accuracy. The tensile strength and impact strength values of the hot-pressed samples were higher than those of the 3D-printed samples, indicating poor adhesion between the printed layers, resulting in voids and points of fracture initiation [40].

### 4.11. Shape Memory Materials

Crosslinking starch-based films with shape memory properties is an alternative to nondegradable heat-shrinkable packaging films. However, the mechanical performance and water resistance of these pure crosslinking starch-based films are poor; thus, one solution is to blend them with PBAT [93]. Long et al. prepared starch/PBAT films by hot pressing and UV crosslinking using benzophenone as a photosensitizer and citric acid as a compatibilizer. Different concentrations (50–90 wt%) of starch were tested. As expected, the tensile strength and elongation at break increased with decreasing starch content. When the PBAT concentration increased, due to the high crosslinking degree, the shape recovery rate increased, reaching a shape recovery ratio of over 90% at 30% pre-tensile strain. Even at a pre-tensile strain of 70%, films with 50 wt% PBAT presented a shape recovery ratio of ~85%. In addition, the films exhibit good water resistance, light transmittance, and biodegradability. These properties make these films suitable for heat-shrinkable packaging applications [93].

### 4.12. Storage Effects on PBAT/TPS Blends

During storage, TPS can recrystallize, which leads to brittleness of the material. When stored above the glass transition temperature, depending on the plasticizer amount, the mobility of the starch chains increases, allowing them to reorganize into more ordered structures. Moreover, TPS absorbs moisture when stored above 50% relative humidity due to its hydrophilicity. The changes in crystallization, which affect mechanical performance, can limit the service life of TPS blends [94]. PBAT can degrade over time, leading to a loss of its mechanical and thermal properties. This can be explained by the carbonyl groups in its chemical structure, which oxidize and generate free radicals that increase the degradation rate of the polymer [34]. Grimaut et al. found that after 1800 days of storage, the tensile strength and elongation at break of PBAT decreased by 73% and 93%, respectively [34]. On the other hand, Geralde et al. found that the flexibility and toughness of PBAT/TPS blends increased with storage time, probably due to the migration of glycerol from TPS into PBAT, plasticizing the continuous phase of PBAT [94].

### 4.13. Microplastics from PBAT/TPS

Even biodegradable materials release microplastics (MPs, particles with a size between 1 µm and 5 mm), generated by the mechanical abrasion of these materials [1,95]. These particles can persist for long periods, possibly releasing additives and allowing them to enter the food chain [96]. Increasing evidence suggests that they tend to bioaccumulate within the food chain, representing a threat to human health [15]. Their toxicity varies according to particle composition, size, and concentration. Because of the degradation properties of biodegradable plastics, which can result in smaller particle sizes, the adverse impacts of MPs derived from these materials on the environment can be higher than that of conventional non-biodegradable plastics [15]. Thus, there are some recent works reporting the effects of starch–PBAT MPs on the soil microbial community, plant growth, soil invertebrates, and aquatic organisms [1,15,95,96,97,98,99].

Most of the recent studies on the effects of starch–PBAT MPs are mulch films composed of this material and compare their effects with those of conventional LDPE, using MP concentrations ranging from typical environmental to the worst-case scenario (0.005–5 wt% of the dry soil weight). Van Loon et al. found that none of the MPs at any concentration posed adverse effects on the survival or reproduction of springtails (*Folsomia candida*) after five generations of exposure [1]. Kokalj et al. also reported similar results for mealworms (*Tenebrio molitor*) at typical environmental concentrations. However, at much higher concentrations of LDPE-MPs, molting and growth of the first generation were reduced, whereas increased growth was observed with starch–PBAT MPs [95]. On the other hand, Smídová et al. found that both types of MPs have some effects on soil properties and negatively affect the reproduction of enchytraeids (*Enchytraeus crypticus*) in the two subsequent generations, with LDPE showing a stronger effect compared to starch–PBAT MPs [96].

Xie et al. investigated the effects of pure PBAT and PBAT/TPS MPs on zebrafish embryos and larvae. When comparing the same concentrations, PBAT/TPS MPs showed a significantly lower impact on the survival rate of zebrafish juveniles and on the hatching rate of embryos than pure PBAT MPs. This can be explained by the starch present in the PBAT/TPS films, which considerably dilutes the concentration of PBAT MPs and is an inert and non-toxic substance. On the other hand, the MPs had no significant effect on the growth and development of the juveniles or on the free-swimming behavior and stimulatory response to light–dark cycle transitions of the larvae [15].

Zantis et al. compared the effects of LDPE and starch–PBAT MPs on barley, wheat, carrot, and lettuce plants by analyzing seed germination and early development (acute effect) and growth and chlorophyll content (chronic effect). It was found that the effects of starch–PBAT MPs were greater than those of LDPE MPs, which is consistent with the few studies comparing the effects of MPs made from biodegradable and non-biodegradable plastics. These findings suggest that further research on the impact of MPs made from biodegradable plastics is urgently needed [99].

## 5. Conclusions

PBAT/TPS blends have been extensively reported in the literature, especially in recent years. This review covers most of these studies, including the compatibilizers and additives used, the various applications of these blends, the starch modifications, the use of chain extenders, and processing methods. This polymer combination is a promising substitute for conventional nondegradable plastics.

However, as shown in this review, most work is focused on the production of films, mainly for food packaging and mulch films for agriculture. Thus, there seems to be a gap that needs to be filled with other applications of PBAT/TPS blends, such as foams and 3D printed materials.

In addition, some improvements can be made, such as increasing the starch content in the blend without significantly affecting the mechanical properties. To this end, there are numerous types and concentrations of compatibilizers and/or additives that can be used, as well as modifications to PBAT or starch that can be made. The impact of the processing conditions and storage time on the properties of the material can also be further studied, as there is little work on this.

Finally, it is necessary to consider the entire life cycle of these blended materials, including their end of life. As described in the section of microplastics, there is insufficient evidence on the safety of MPs derived from these PBAT/TPS materials. Therefore, a more detailed analysis of their toxicity to the environment is still pending.

## Data Availability

Data will be made available on request.
